# Cultivated *Escherichia coli* diversity in intestinal microbiota of Crohn's disease patients and healthy individuals: Whole genome data

**DOI:** 10.1016/j.dib.2019.104948

**Published:** 2019-12-05

**Authors:** Maria Siniagina, Maria Markelova, Alexander Laikov, Eugenia Boulygina, Dilyara Khusnutdinova, Anastasia Kharchenko, Albina Misbakhova, Tatiana Grigoryeva

**Affiliations:** a Kazan Federal University, Russian Federation; b Kazan Medical State University, Russian Federation

**Keywords:** *Escherichia coli*, Crohn's disease, Whole-genome sequencing, Human gut microbiota

## Abstract

Dysbiosis of the gut microbiota in inflammatory bowel disease (IBD) patients is of great interest. It has been reported that Crohn's disease (CD) is associated with a general decrease in microbial diversity [1]. Altered microbial composition and function in CD results in imbalance in host-bacteria interaction and increased immune stimulation [2]. It is shown that microbiota in CD is characterized by increased proportion of *E. coli* in human gut in contrast to healthy individuals [3]. However, the overall qualitative and quantitative diversity of *E. coli* strains in CD is not fully understood. Here, we present a dataset of whole-genome sequences of *E. coli*'s.

**Table:** Specifications Table

Subject	Immunology and microbiology
Specific subject area	Microbiology
Type of data	Whole-genome sequencing data, table, figure
How data were acquired	Whole-genome sequencing on Illumina MiSeq platform. Bioinformatics approaches: genome assembler SPAdes v.3.11.1, rapid prokaryotic genome annotation Prokka v.1.12, pan genome Roary pipeline v.3.12.0, FastTree v.2.1.11 tool, SerotypeFinder-2.0 tool.
Data format	Raw, analyzed, deposited data
Parameters for data collection	Whole genomes of *E. coli* isolates from patients with diagnosed Crohn's disease and healthy individuals were sequenced, assembled and annotated
Description of data collection	Dataset covers 64 samples (*E. coli* isolates from stool samples of 18 healthy individuals and 14 Crohn's disease patients)
Data source location	Kazan Federal University, Kazan, Russian Federation
Data accessibility	The whole genome sequencing data have been deposited to NCBI BioProject with the dataset identifier PRJNA560176 https://www.ncbi.nlm.nih.gov/bioproject/560176
Related research article	Miquel S., Peyretaillade E., Claret L., De Vallée A., Dossat C., Vacherie B., Zineb E., Segurens B., Barbe V., Sauvanet P., Neut C., Colombel, J., Medigue C., Mojica F., Peyret P., Bonnet R., Darfeuille-Michaud A. Complete genome sequence of Crohn's disease-associated adherent-invasive *E. coli* strain LF82, PloS one, 5(9) (2010), p. e12714, https://doi.org/10.1371/journal.pone.0012714

**Table:** 

**Value of the Data** • The sequence data will be useful for comparative genomic and transcriptomic studies of *E. coli* to discover the genetic determinants which may be related to Crohn's disease (CD). • The complete genome sequences of *E. coli* strains isolated from patients with CD and healthy individuals provide data about frequency of occurence of virulence and pathogenic factors in human gut microbiome. • *In silico* serotyping can be useful in studies on interaction between the host immune system and *E. coli* in CD.

## 1 Data

Previous studies showed that CD patient's immune system has aberrant response to gut microbiota resulting in decreased bacterial diversity accompanied by enrichment of Enterobacteriaceae family [1], [2], [3].

In the present article, we report whole genome data of cultivated *E. coli* strains isolated from stool samples of 14 CD patients and 18 controls (listed in Supplementary Table 1). Out of 97 sequenced genomes, 33 duplicates were revealed using the comparative genome analysis, i.e. isolates sequenced more than once due to varying colony phenotypes. Thus, 64 unique *E. coli* genomes were obtained: 27 from CD patients (6 from patients with diagnosed ileitis, 14 – colitis, 7 – ileocolitis), and 37 from the control group (Supplementary Table 2). *E. coli* draft genome assemblies were submitted to NCBI (BioProject ID PRJNA560176).

Phylogenetic group analysis, performed according to Clermont [4], revealed that *E. coli* strains of E and F groups were observed only in healthy donors.

**Fig. 1:**
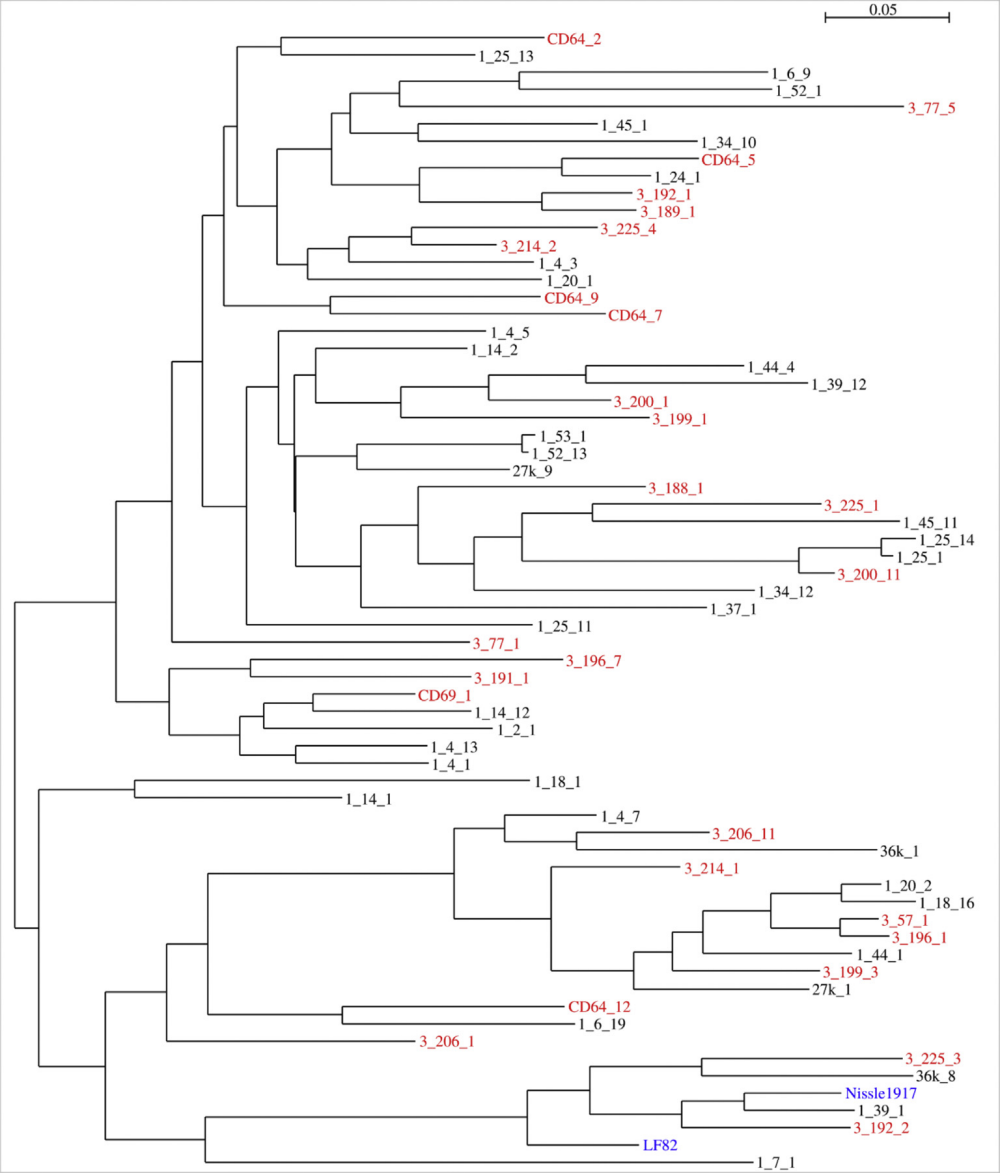
Phylogenetic tree of *E. coli* strains from CD patients (red), healthy individuals (black) and reference genomes (blue) based on accessory gene content in genome assemblies.

**Fig. 2:**
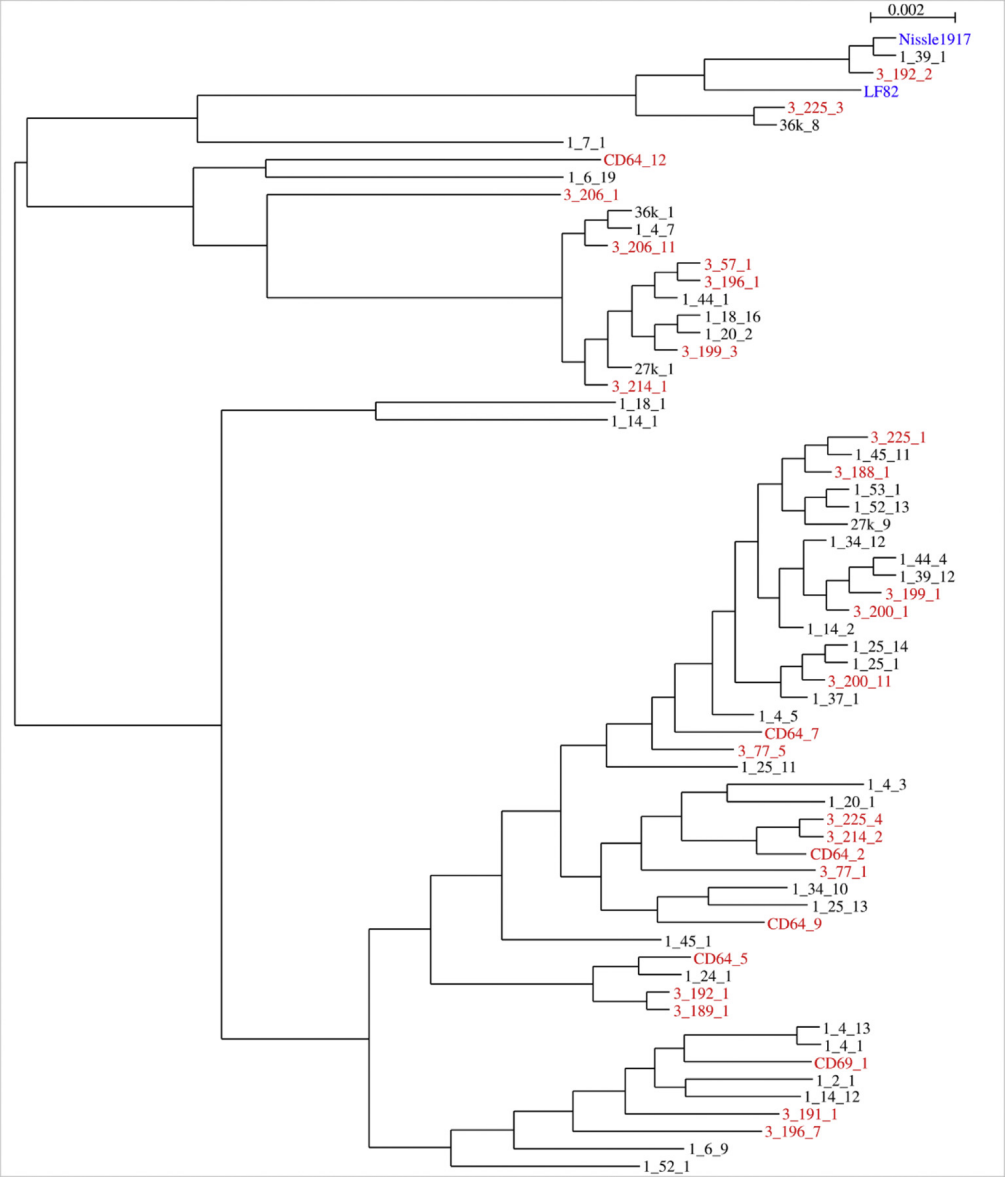
Phylogenetic tree of *E. coli* strains from CD patients (red), healthy individuals (black) and reference genomes (blue) based on core genes in genome assemblies.

Phylogenetic trees analysis based on core and accessory genes did not reveal any specific *E. coli* group associated with the disease. For comparison LF82 strain associated with ileal CD [5] and widely studied probiotic strain Nissle 1917 [6] were included as references (Fig. 1, Fig. 2).

**Fig. 3:**
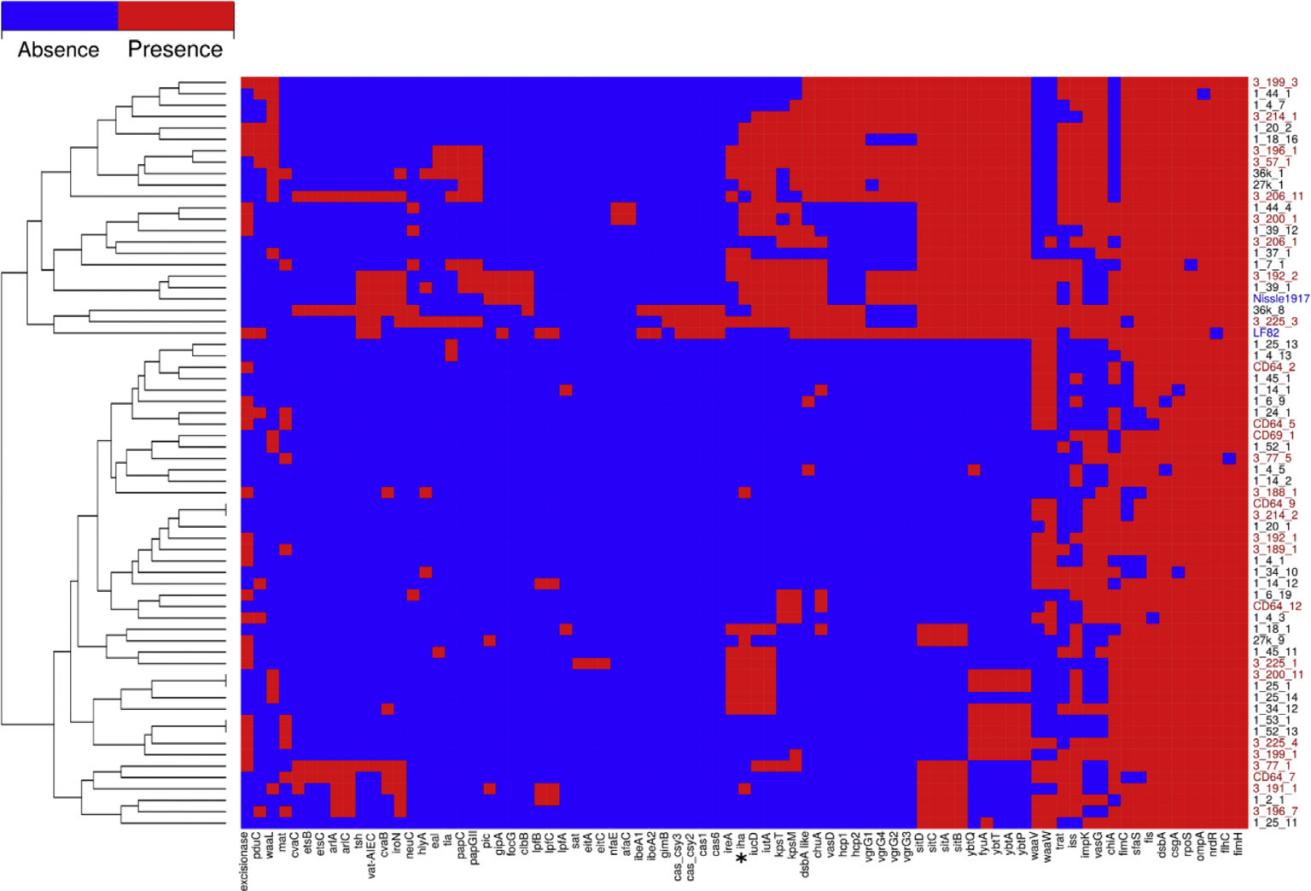
Distribution of virulence and pathogenicity genes of *E. coli* from CD patients (red), healthy individuals (black) and reference strains LF82 and Nissle 1917 (blue). Genes present or absent in all analyzed strains are not displayed. Gene with differential distribution in strains from patients with ileitis vs patients with colitis and ileocolitis is marked with asterisk (*).

Analysis of 98 previously reported genes associated with pathogenicity and virulence in *E. coli* [7,8] revealed that the frequency of occurrence of *iha* gene coding bifunctional enterobactin receptor/adhesin protein among strains from patients with ileitis was higher than with colitis and ileocolitis (exact Fisher test, P = 0.044 with, P value with Benjamini-Hochberg adjustment) (Fig. 3).

*In silico* serotyping showed a vast diversity of *E. coli* serotypes in both studied cohorts. However, no serotype associated with the disease was found. Strains of 5 serological types were represented both in CD group and control one - O17/O44:H18, O144:H45, O6:H1, O25:H18, O1:H7.

## 2 Experimental design, materials, and methods

### 2.1 Sample collection

A total of 32 stool samples, 14 from patients with Crohn's disease diagnosed by colonoscopic examination and confirmed histologically, and 18 from healthy individuals were taken for the analysis. The samples were collected at the Kazan Federal University Hospital (Kazan, Russia) and stored at −80 °C until needed.

### 2.2 Isolation and identification of *E. coli* strains

Serial ×10 fold dilutions in PBS solution were made from 0.1 g of stool sample. 0.1 ml of suspension (×10^2^–10^3^ fold) was poured onto Endo agar medium and incubated at 37 °C for 19–20 hours. The total number of colonies was counted and colony morphology (color, shape, size, metallic luster) was registered. Up to 10 representative from each sample lactose-positive colonies (dark red color) were randomly picked up for cultivation in LB medium at 37 °C for 19–20 hours. The identification of the *E. coli*-like colonies was confirmed using MALDI Biotyper System (Bruker, Germany). Lactose-negative colonies after testing against polyvalent anti-Shigella sera were added to the collection for further sequencing (Agnolla, Russia). In addition, the ability to hemolyze red blood cells was assessed by the presence of clear zones around colonies on blood agar medium after 24 hours of incubation at 37 °C. Relative and absolute abundances of isolated strains are represented in Supplementary Table 2. The mean CFU/g of feces from healthy individuals and CD patients were 3.4*10^5^ and 3.8*10^5^, respectively (one strain with extremely high abundance was excluded).

In total 521 isolates were collected and stored in tryptic soy broth containing 50% glycerol at −80 °C until further phylotype screening.

### 2.3 DNA extraction and *E. coli* phylotyping

Genomic DNA was extracted from colonies with PureLink Genomic DNA Mini Kit (Invitrogen) following the manufacturer instructions and quantified using Qubit 2.0 Fluorometer (Invitrogen). The *E. coli* phylogroup (A, B1, B2, C, D, E, F) of each colony was determined by the quadruplex PCR [4].

### 2.4 Genome sequencing and analysis

Selected 97 isolates assigned to different phylogenetic groups and/or morphology were subjected to the whole-genome sequencing. DNA libraries were prepared using NEBNext Ultra II Kit (New England BioLabs, USA) according to the manufacturer's recommendations. DNA-library size was evaluated on the Agilent 2100 Bioanalyzer (Agilent Technologies, USA). The sequencing was performed on Illumina MiSeq platform (300 bp paired-end mode).

After adapters removal and filtering by length and quality using cutadapt [9] paired-end reads were *de novo* assembled using SPAdes v.3.11.1 (http://cab.spbu.ru/software/spades/) [10]. Genome annotation was performed using Prokka v.1.12 [11] and pangenome analysis was performed with Roary pipeline v.3.12.0 [12]. Phylogenetic trees based on core and accessory genes was constructed using FastTree v.2.1.11 [13]. Serotypes were assigned using SerotypeFinder-2.0 tool [14].
